# ^18^F-FDG–Avid Axillary Lymph Nodes After COVID-19 Vaccination

**DOI:** 10.2967/jnumed.121.262108

**Published:** 2021-10

**Authors:** B. Jake Johnson, Kathryn M. Van Abel, Daniel J. Ma, Derek R. Johnson

**Affiliations:** * Mayo Clinic Rochester, Minnesota

**FIGURE 1.**
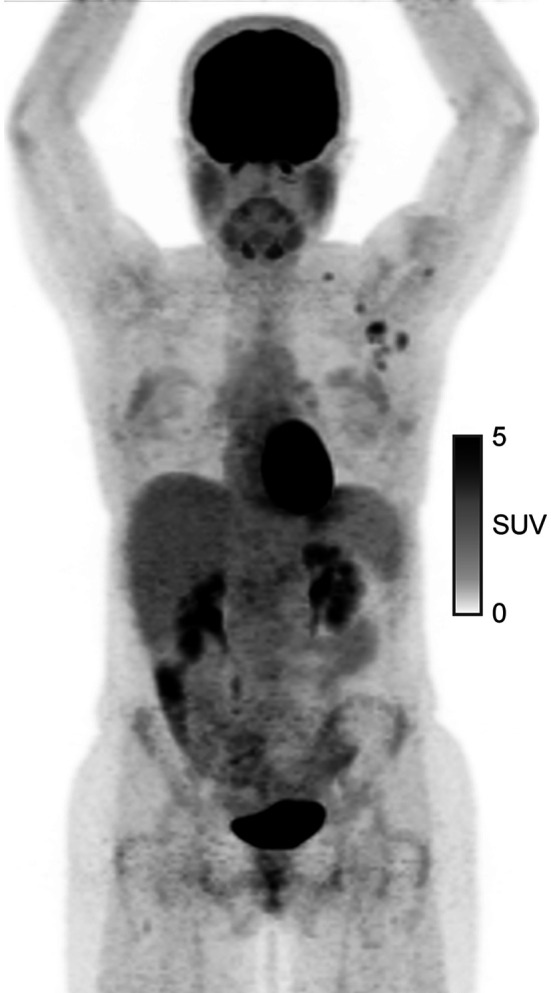
^18^F-FDG PET/CT showing left axillary and left supraclavicular avidity. Maximum-intensity-projection image with SUV scale at right.

**TO THE EDITOR:** In a recent patient with a left-side parotid malignancy (biopsy-proven mammary analog secretory carcinoma), ^18^F-FDG PET/CT was obtained during the workup (Fig. 1). The findings showed ^18^F-FDG avidity in the left axillary lymph nodes with an overall SUV_max_ of 4.5 and an ^18^F-FDG–avid left supraclavicular lymph node. This result prompted an ultrasound-guided biopsy of the lymph nodes before surgery. Pathologic examination of both subsites revealed lymphocytes consistent with a benign lymph node. Around the time of the biopsy, the patient recalled that she had received the first dose of the Moderna Therapeutics messenger RNA-1273 vaccine 10 d beforehand in her left deltoid. After vaccination, she had injection site soreness and some mild fatigue and general malaise for about 4 h. She then underwent successful superficial parotidectomy, with margin-negative and node-negative resection of the left parotid mammary analog secretory carcinoma.

Shortly after the aforementioned patient was seen, 3 mo posttreatment PET imaging was obtained as part of oncologic surveillance for a patient with a history of oral cavity/oropharyngeal squamous cell carcinoma. On physical examination 3 d before her PET study, laryngoscopy revealed findings concerning for recurrence in the previous surgical bed. Both sides of the neck were palpated, and no lymphadenopathy was appreciated. On PET, the left axillary and left supraclavicular nodes had ^18^F-FDG avidity, with an SUV_max_ of 5.1. Because of our previous experience with the other patient, this second patient was questioned specifically regarding coronavirus disease 2019 (COVID-19) vaccination. She was able to recall that she had received the first dose of the COVID-19 vaccine 14 d beforehand, though she could not recall the manufacturer. The patient reported minimal symptoms after vaccination and was asymptomatic at the time of the PET scan. She was taken to the operating room for direct laryngoscopy, and biopsy of the concerning area revealed mild dysplasia with no evidence of carcinoma.

^18^F-FDG uptake is not tumor-specific and can be seen in infection, inflammation, and granulomatous disease (*1*). Axillary lymph node ^18^F-FDG avidity has been reported in patients receiving several types of vaccines, including vaccinations to influenza, H1N1, and the human papillomavirus vaccine, but has not been reported in association with the COVID-19 vaccine (*2*–*4*). Ultrasound-guided fine-needle aspiration is generally a low-morbidity procedure, though no procedure is without risk. Biopsy of her axillary node could likely have been avoided if the correlation between her recent history of vaccination and her left axillary ^18^F-FDG–avid lymph nodes had been determined. Limited data on mammary analog secretory carcinoma shows a 5.5% rate of cervical nodal metastasis, but biopsy of a supraclavicular node with ^18^F-FDG uptake is prudent in the setting of an ipsilateral parotid malignancy (*5*).

As vaccination against the 2019 novel coronavirus becomes more widespread, it will be important to consider vaccination history, especially in patients who undergo ^18^F-FDG PET/CT for cancer staging or surveillance. Reporting the vaccine history and injection location before obtaining PET imaging may help with interpretation of these studies. Further study could reveal what percentage of patients have ^18^F-FDG–avid lymph nodes after vaccination and elucidate the time required after vaccination to allow for resolution of uptake in regional lymph nodes. This information may be able to guide recommendations on the timing of PET imaging and COVID-19 vaccination.
